# Mortality and Institutionalization After a Potentially Preventable Hospitalization Among People with Dementia

**DOI:** 10.1093/geroni/igaf122.169

**Published:** 2025-12-31

**Authors:** Elham Mahmoudi

**Affiliations:** University of Michigan, Ann Arbor, Michigan, United States

## Abstract

Potentially preventable hospitalizations (PPHs) are costly and harmful to all older adults. There is a paucity of evidence on adverse health events of PPH among patients with dementia. We examined the effect of any PPH in 2018 on mortality and long-term institutionalization in 2019 among Medicare beneficiaries with dementia. We used 20% random sample of 2018-2019 Medicare claims data to select people with dementia who continuously enrolled in 2018 and 2019 traditional Medicare with at least one hospitalization in 2018 (n = 67,110). We also identified people with at least one PPH in 2018 (20.1% of the sample). We used the average treatment effect on treated method, matching the characteristics of people with and without PPH and applying robust standard errors. Our models controlled for age, sex, race/ethnicity, frailty index, rurality, neighborhood disadvantaged index, and number of primary care providers and hospitals at the county level. The average effect of at least one PPH on mortality was 33.5% (95% CI: 32.7%-34.3%) among people with PPH compared to 28.8% (95% CI: 28.3%-29.2%) among those with no PPH. Thus, among dementia patients, a PPH increased the risk of mortality by 4.7 percentage points (PP) (95% CI: 3.8%-5.6%). Effect of any PPH on long-term institutionalization was 21.1% (95% CI: 20.3%-22.0%) among people with PPH compared to 17.9% (95% CI: 17.4%-18.3%) among those with no PPH. Having any PPH increased the risk of long-term institutionalization by 3.3 PP (95% CI: 2.3%-4.2%). PPHs increase the risks of mortality and long-term institutionalization among older adults with dementia.

